# Increase of Antitumoral Effects of Cytokine-Induced Killer Cells by Antibody-Mediated Inhibition of MICA Shedding

**DOI:** 10.3390/cancers12071818

**Published:** 2020-07-07

**Authors:** Xiaolong Wu, Ying Zhang, Yutao Li, Ingo G.H. Schmidt-Wolf

**Affiliations:** Department of Integrated Oncology, CIO Bonn, University Hospital Bonn, D-53105 Bonn, Germany; Xiaolong.Wu@ukbonn.de (X.W.); Ying.Zhang@ukbonn.de (Y.Z.); Yutao.Li@ukbonn.de (Y.L.)

**Keywords:** cytokine-induced killer (CIK) cells, MICA/B, NKG2D, shed MICA, monoclonal antibody

## Abstract

Natural killer group 2D (NKG2D) receptor plays a pivotal role in cytokine-induced killer (CIK) cell-mediated cytotoxicity against malignancies, and the expression of NKG2D ligands might allow targets to be more susceptible to the CIK cell-mediated destruction. In this study, we investigated the synergistic effects of CIK cells antitumor activity and antibody-mediated inhibition of MICA/B shedding. This monoclonal antibody (7C6) has been previously shown to be able to specifically target MICA/B a3 domain on tumor cells, resulting in the increase in cell surface MICA/B expression by inhibition of their shedding. In the current study, we show that 7C6 antibody could substantially inhibit MICA shedding and stabilize the expression of MICA/B on Hela cells and MDA-MB-231 cells. In combination with 7C6, CIK cells showed higher degranulation rate, more IFN-γ production and elevated cytotoxic capacity against tumor cells. Furthermore, we demonstrate that NKG2D-MICA/B ligation could lead to activation of both CD3+ CD56− T cells and CD3+CD56+ NKT subset cells of CIK culture and NKT subset was more sensitive to NKG2D signaling than the counterpart T cells. 7C6-mediated inhibition of MICA shedding could strengthen this signal and eventually enhance the antitumor activity of CIK cells. With multiple advantages of easy ex vivo expansion, minor GVHD, natural tumor trafficking and non-MHC restricted, CIK cell-based therapy may serve as a potent combination partner with MICA antibody-mediated immunotherapy.

## 1. Introduction

Immunotherapy is emerging as a revolution in cancer treatment. Chimeric antigen receptor (CAR)-T cell therapy has shown clinical success in the treatment of certain blood cancers and has been approved by the FDA. However, this therapy is so far limited in hematological malignancies with potentially fatal treatment-related side effects, such as cytokine release syndrome (CRS) and neurotoxicity. Apart from those, the expensiveness and complex in genetical modification and expansion of this product may leave a hurdle in its broad investigation and application. Therefore, other alternatives are needed to join in this long-term battle. Cytokine-induced killer (CIK) cells are a heterogeneous population expanded ex vivo which share the phenotypic and functional properties of both NK and T cells [1]. With a broad antitumor spectrum, CIK cells have been shown to have therapeutic effect in patients with both hematological and solid cancers [2,3]. Due to the easy ex vivo expansion, minor GVHD, natural tumor trafficking and non-MHC restricted, intensive attempts have been made in an effort to improve the therapeutic efficacy of CIK cells in both basic and clinical research since its first report [4,5,6,7,8].

CIK cell expansion typically takes two to three weeks in the presence of a cocktail of stimuli, IFN-gamma, anti-CD3 Ab, IL-β1 and IL-2. Afterwards, a substantial increase in both percentage and absolute number of CD3+ CD56+ “NKT” phenotyping population can be obtained. This subset accounts for the main cytolytic population in bulk CIK culture [9]. Various molecules have been found to be involved in the antitumor activity of CIK cells, such as adhesion molecule lymphocyte function associated antigen-1 (LFA-1) [10], NK cell activating receptors-NKG2D, NKp30, CD16 and DNAX accessory molecule-1 (DNAM-1) [11,12,13]. TCR/CD3 complex (MHC-restricted manner) [11], program cell death system FasL and Trail signaling [14,15], granules perforin and granzymes [10]. Among them, NKG2D has been shown to play a vital role in the antitumor activity of CIK cells.

NKG2D, a C-type lectin surface receptor, is known as an activating receptor which can stimulate NK cells, γδ T cells and co-stimulate CD8αβ T cells [16,17]. In human, NKG2D receptor recognizes a broad range of ligands, including family of MHC I Chain-related molecules A and B (MICA and MICB), and family of six cytomegalovirus UL16-binding proteins (ULBP1-6) [18,19]. These ligands are generally absent on normal cells with the exception of gastrointestinal epithelium, but are often inducible under certain stressed conditions, for example, viral infection and tumorigenic transformation. Expression of NKG2D ligands on tumor cells render them more sensitive to immunological destruction by engaging NKG2D receptor to trigger NK cells, γδ T cells and provide costimulatory signal for CD8 T cells [16,17]. MICA and MICB are the best characterized and most prevalently expressed ligands by human tumors [20]. However, several molecules have been shown to be associated with the proteolytic shedding of MICA and MICB, leading to the immune escape in advanced cancers, such as disulfide isomerase (ERp5) and ADAM (a disintegrin and metal-loproteinase) proteins and MMPs (matrix metalloproteinases) [21,22,23,24]. Recently, a new monoclonal antibody which is able to specifically target the MICA a3 domain could lead to the inhibition of MICA/B shedding and the stabilization of surface MICA/B expression [25]. In this study, our aim is to investigate the synergistic antitumor effects of CIK cells and 7C6 antibody-mediated inhibition of MICA shedding.

## 2. Results

### 2.1. NKG2D Expression and Phenotype of CIK Cells

**Figure 1:**
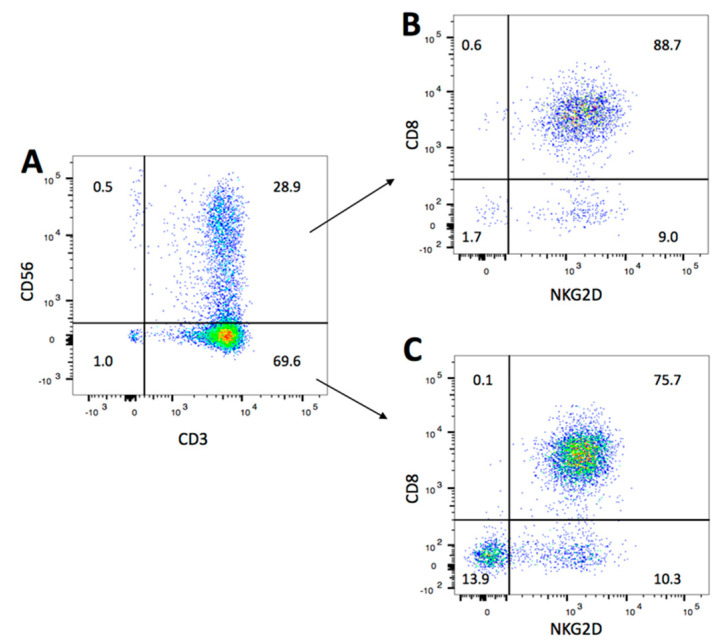
Phenotyping and NKG2D expression on CIK cells. PBMC was isolated from heathy donors and cultured in the presence of IFN-γ on day 0, anti-CD3, IL-2, and IL-1β on day 1. Cells were subcultured with IL-2 and fresh medium every three days. After 14 days of expansion, phenotype and NKG2D expression of CIK cells were measured on flow cytometry after staining with FITC-CD3, PE-CD56, BV421-CD8 and APC-NKG2D antibodies. Dead cells were excluded by 7AAD. (**A**) The percentage of various subsets of bulk CIK cells. (**B**) The expression of CD8 and NKG2D within CD3+CD56+ population. (**C**) The expression of CD8 and NKG2D within CD3+CD56- population. Numbers represent the percentage (%) of individual gating. Data are one representative of CIK cells from four donors.

After 14 days of ex vivo expansion, the bulk CIK cells were a heterogeneous population composed by CD3+CD56+ “NKT” cells (31.3% ± 11.2%), CD3+CD56− T cells (65.6% ± 8.9%), and a small percentage of CD3-CD56+ NK cells (2.8% ± 4.4%) (Figure 1A, one representative data of four donors). In particular, the percentage of CD8+ component of CD3+CD56+ “NKT” cells was slightly higher than that of CD3+CD56− T cells (83.0% ± 12.4% vs 79.5% ± 5.7%, respectively). At day 14, most of cells expressed NKG2D (90.7% ± 2.0%). As shown in Figure 1B and Figure 1C, CD8+ cells both within “NKT” cells and T cells were the main cells with NKG2D expression. These data are consistent with early reports by our and others [26,27].

### 2.2. Inhibition of MICA Shedding and Stabilization of Surface MICA/B Expression on Tumor Cells by 7C6 Antibody

**Figure 2:**
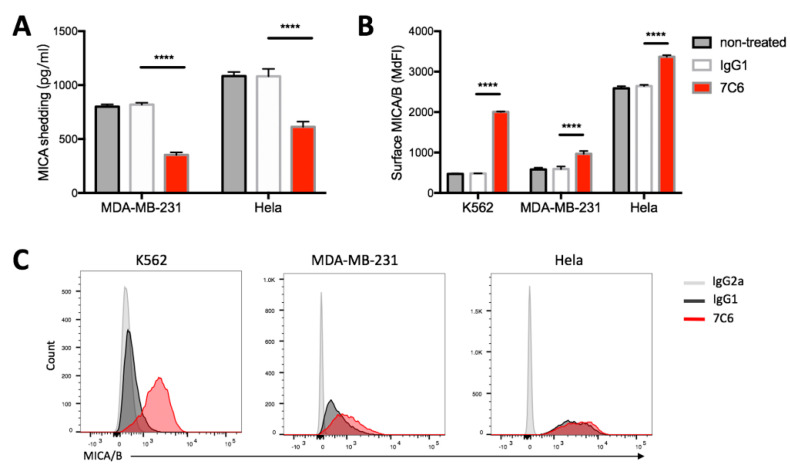
7C6 antibody inhibits MICA shedding and increases the cell surface density of MICA/B. (**A**) Human Hela cervical and MDA-MB-231 breast cancer cell lines were untreated or treated with 7C6 or IgG1 control antibody at 10 µg/mL for 48 h. Shed MICA was quantified in the supernatant by sandwich ELISA. Data are mean ± SD of triplicate measurements; data are one representative of three independent experiments. **** *p* < 0.0001 calculated by two-way ANOVA, Bonferroni’s post-hoc test. (**B**) K562, Hela, and MDA-MB-231 cells were treated with 7C6 or IgG1 control antibody at 10 µg/mL for 24 h. Surface MICA/B expression was measured by flow cytometry following staining with APC-conjugated 6D4 antibody; median fluorescence intensities (MdFI) are shown. Data are mean ± SD of triplicate measurements; data are one representative of three independent experiments. **** *p* < 0.0001 calculated by two-way ANOVA, Bonferroni’s post-hoc test. (**C**) Histograms depict the surface level of MICA/B following treatment with 7C6 (red) or IgG1 control (black). IgG2a (grey) was the staining isotype control. Data are part of the experiment shown in ‘B’.

Compared with the control IgG1 antibody treatment, the MICA shedding from Hela cells in the presence of 7C6 antibody was strongly inhibited (Figure 2A, 1081.9 ± 69.1 pg/mL vs. 613.4 ± 48.6 pg/mL, respectively) and the surface MICA/B expression was significantly increased (Figure 2B, MdFI, 2644.5 ± 29.0 vs. 3367.7 ± 40.7, respectively). Similar results were observed when MDA-MB-231 cells used as targets, less shedding of MICA (Figure 2A, 819.6 ± 17.6 pg/mL vs. 352.8 ± 24.7 pg/mL, respectively) and increased surface MICA/B expression (Figure 2B, MdFI, 592.3 ± 64.0 vs. 968.7 ± 70.2, respectively) were shown in the presence of 7C6 antibody. As shown in Figure 2B,C, there was nearly a three-fold increase in the MICA/B expression on K562 cells after treatment with 7C6 antibody versus IgG1 control. However, no MICA shedding from K562 cells was detected (Figure S1). As reported by others [28], K562 cells mainly shed MICB. Thus, the increase in surface MICA/B expression following 7C6 antibody treatment is more likely led by the inhibition of MICB shedding. Consistent with the previous study [25], our data demonstrate that 7C6 antibody is able to strongly inhibit the proteolytic shedding of MICA from MICA/B-bearing tumor cells, resulting in a substantial increase in the cell surface density of MICA.

### 2.3. 7C6 mAb Enhances the In Vitro Antitumor Activity of CIK through the NKG2D-MICA/B Axis

**Figure 3:**
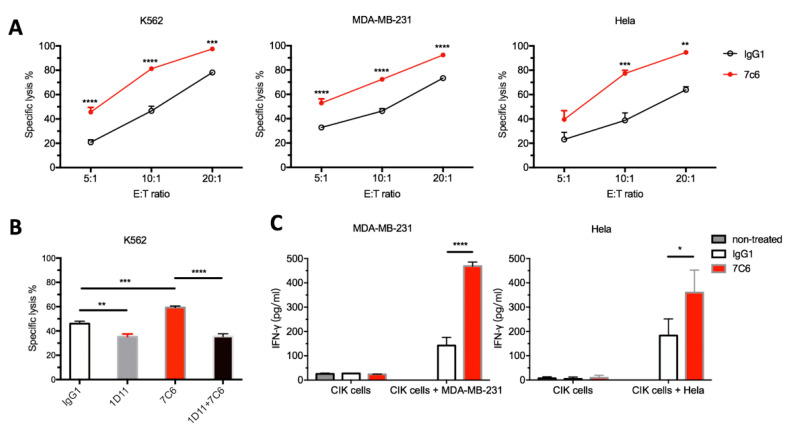
7C6 mAb increases cytotoxicity and cytokine production of CIK cell. After 14 days of ex vivo expansion in the presence of IL-2, CIK cells were harvested and co-cultured with indicated tumor cells. 7C6 or isotype control IgG1 antibody was added to co-culture at a concentration of 10 µg/mL. (**A**) The indicated tumor cells were used as target cells for CIK cell-mediated lysis in FACS-based cytotoxicity assay. Data are mean ± SD of triplicates per condition and one representative of three independent experiments. ** *p* < 0.01, *** *p* < 0.001, **** *p* < 0.0001 calculated by two-way ANOVA, Bonferroni’s post-hoc test. (**B**) CIK cells were pretreated with NKG2D blocking 1D11 antibody or IgG1 control antibody at 10 µg/mL 1 h before coculture with CFSE labelled K562 cells at 10:1 E/T ratio in the presence of 7C6 or IgG1 control antibody at 10 µg/mL. After 20 h, cytotoxicity was determined by FACS-based assay. Data are mean ± SD of triplicates per condition and one representative of three independent experiments. ** *p* < 0.01, *** *p* < 0.001, **** *p* < 0.0001 calculated by one-way ANOVA, Bonferroni’s post-hoc test. (**C**) Following co-culture with tumor cells for 24 h at a 20:1 of E:T ratio, IFN-gamma production in the supernatant was detected by sandwich ELISA. Data are mean ± SD of triplicates per group, representative of three independent experiments. * *p* < 0.01, **** *p* < 0.0001 calculated by two-way ANOVA, Bonferroni’s post-hoc test.

As shown in Figure 3A, the cytolytic ability of CIK cells was significantly enhanced in the presence of 7C6 mAb against indicated tumor targets, as compared to the IgG1 control treatment. For example, 81.3 ± 3.4% vs 46.6 ± 6.7% of K562 cells, 72.3 ± 1.0% vs. 46.3 ± 3.6% of MDA-MB-231 cells and 77.4 ± 4.6% vs. 38.8 ± 10.7% of Hela cells were killed in each corresponding E/T coculture at a 10:1 E/T ratio. However, this enhancement in CIK cell killing was completely inhibited to the same extent as that in the presence of IgG1 isotype antibody when CIK cells were pretreated with NKG2D blocking antibody (Figure 3B).

In addition, stimulation by tumor cells led to substantial cytokine release by CIK cells, as shown in Figure 3C. The production of IFN-gamma could be further dramatically augmented after 24 h of MDA-MB-231/CIK coculture in the presence of 7C6 mAb as compared with isotype IgG1 (469.1 ± 16.6 pg/mL vs. 142.3 ± 33.8 pg/mL, respectively). Similar results were found in Hela/CIK coculture, 359.6 ± 93.3 pg/mL vs. 183.0 ± 68.6 pg/mL, respectively.

Taken together, these data indicate that 7C6 mAb can promote the CIK-mediated cytotoxicity against these tumor targets, through the NKG2D-MICA/B pathway.

### 2.4. NKG2D-MICA/B Ligation Leads to the Triggering and Activation of both CD3+CD56+ NKT Cells and CD3+CD56− T Subset of CIK Cells

**Figure 4:**
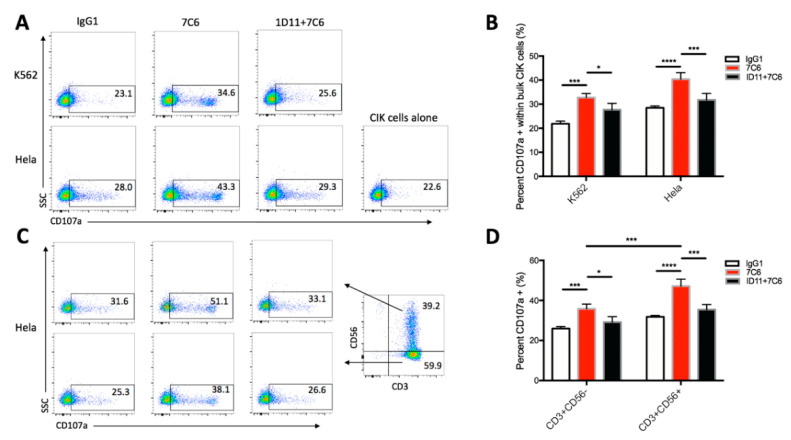
7C6 mAb increases the degranulation of CIK cells, both CD3+CD56+ NKT cells and CD3+CD56- T cells against tumor targets. CIK cells were pretreated with 1D11 or IgG1 antibody at 10 µg/mL 1 hour prior to coculture with tumor cells. Afterwards, pretreated CIK cells were co-incubated with indicated tumor targets at 5:1 E/T ratio in the presence of 7C6 mAb or IgG1 antibody at 10 µg/mL. APC-CD107a was added at the start of coculture. At the end of 4 h co-incubation, degranulation of CIK cells was determined using flow cytometry by staining cells with FITC-CD3 and PE-CD56 antibodies. (**A**) The degranulation of bulk CIK cells against K562 cells (upper panel) and Hela cells (lower panel) in the presence of indicated antibodies is shown in dot plot. Numbers represent the percentage of gated population. (**B**) Data are mean ± SD of triplicates from ‘A’, representative of three independent experiments. * *p* < 0.05, *** *p* < 0.001, **** *p* < 0.0001 calculated by two-way ANOVA, Bonferroni’s post-hoc test. (**C**) The degranulation of CD3+CD56+ (upper panel) and CD3+CD56− (lower panel) subset cells were further analyzed from the same data set in ‘A’. Numbers represent the percentage of gated population. (**D**) Data are mean ± SD of triplicates from ‘C’, representative of three independent experiments. * *p* < 0.05, *** *p* < 0.001, **** *p* < 0.0001 calculated by two-way ANOVA, Bonferroni’s post-hoc test.

To investigate the role of NKG2D in activating CIK cells, we performed the degranulation assay in which CD107a has been widely used as a marker for activated cytotoxic lymphocytes, both CD8+ T cells [29] and NK cells [30]. In Figure 4A, both K562 cells and Hela cells cultured in the presence of 7C6 mAb were shown to remarkably upregulate the degranulation of bulk CIK cells in contrast to the IgG1 treatment (Figure 4B, 32.8 ± 1.7% vs. 21.8 ± 1.1%, 40.4 ± 2.7% vs. 28.5 ± 0.8%, respectively). However, masking CIK cells with 1D11 blocking antibody significantly reduced the upregulation in degranulation compared to 7C6 treatment (Figure 4B, 27.7 ± 2.6% vs. 32.8 ± 1.7%, 31.8 ± 2.7% vs. 40.4 ± 2.7%, respectively), close to that with treatment of IgG1 control alone. Similar effects were also observed when the CD3+CD56+ and CD3+CD56− subsets were further analyzed. As shown in Figure 4C,D and Figure S2, both subsets showed significant increase in degranulation after treatment with 7C6 mAb versus Hela cells (31.8 ± 0.7% vs. 47.2 ± 3.4%, 26.0 ± 1.0% vs. 35.8 ± 2.4%, respectively) and K562 targets (22.5 ± 0.9% vs. 39.4 ± 1.4%, 20.9 ± 1.4% vs. 28.3 ± 1.9%, respectively) while decrease after 1D11 antibody blocking against Hela cells (47.2 ± 3.4% vs. 35.4 ±2.6%, 35.8 ± 2.4% vs. 29.2 ± 2.8%, respectively) and K562 cells (39.4 ± 1.4% vs. 29.4% ± 2.0%, 28.3 ± 1.9% vs. 26.2 ± 3.0%, respectively). Of note, CD3+CD56+ cells exhibited a higher response to the treatment of 7C6 mAb than CD3+CD56− subset against Hela cells (Figure 4D, 47.2 ± 3.4% vs. 35.8 ± 2.4%, respectively) and against K562 cells (Figure S2B, 39.4 ± 1.4% vs. 28.3 ± 1.9%, respectively).

Altogether, these data demonstrate that NKG2D-MICA/B ligation can lead to the triggering and activation of both CD3+CD56+ and CD3+CD56− subsets of CIK cells. 7C6 mAb-mediated MICA/B stabilization is able to enhance the activation of CIK cells.

## 3. Discussion

CIK-based therapy has been approved to treat patients with hematological malignancies at high risk of relapse after allogeneic transplantation in Germany since 2014 (national authorization § 4b Abs. 3 AMG; “Hospital Exemption”). Recently, Michael Merker et al. [31] found that T cell recovery was significantly improved after CIK cell therapy in patients with relapsing hematological malignancies after allogeneic hematopoietic stem cell transplantation (HSCT). Other studies have also shown improved overall survival and progress-free survival in patients with solid malignancies after the treatment of CIK cells [3,32]. However, tumors seem to always be able to find ways to escape from the surveillance of host immune system. MICA/B shedding is one of the contributors. Therefore, multi-therapeutic regimes in combination would provide more benefits for cancer patients.

In the current study, a novel antibody which has been developed to specifically target α3 domain of MICA and MICB molecules while sparing the NKG2D binding site α1α2 domain was used [25]. In agreement with the previous report [25], our study show that anti-MICA specific antibody could strongly inhibit the loss of MICA from the tumor membrane surface and substantially increase the expression of MICA/B. As they proposed, this might be the consequence of the competitive binding of MICA antibody to the α3 domain which could leave less space for ERp5 and protease to execute the shedding.

NKG2D could be induced in the presence of IL-2, regardless of the dosage, along with the ex vivo CIK expansion, but only cells cultured in high doses of IL-2 gained expression of the adapter protein DAP10 and were capable of cytotoxic function [12]. In this study, we show that the CD3+CD56+ NKT cell population has the highest expression of NKG2D, this may facilitate it to be the main killer in the bulk CIK cells. In the presence of 7C6 antibody, a significant increase in cytolytic capability, IFN-gamma production and degranulation of CIK cells was observed against tumor cells as compared to IgG1 control antibody. However, the effect on the augmentation in cytotoxicity and degranulation was nearly completely inhibited by pretreatment of CIK cells with NKG2D blocking antibody. These indicate no or little involvement of other molecules except for the NKG2D receptor in the 7C6-driven enhancement of CIK cell killing against target cells. Although 7C6 mAb was shown to be able to induce the ADCC (antibody-dependent cellular cytotoxicity) effect in NK cells by engaging the CD16 Fc receptor [25], it seemed no engagement of CD16 occurred in our study. Of course, the data shown here can only represent the CIK cells (from 10 donors) cultured in this study. We cannot conclude that there is no engagement of CD16 on all CIK cells in general. Because CD16 expression on CIK cells was recently shown to be donor-dependent and could mediate the ADCC activity [13], although it was early reported to be mostly absent [9]. ADCC could be induced by 7C6 mAb if a certain amount of CD16 is expressed on CIK cells, but this needs further investigation.

It has been clear that CD3+CD56+ subset has a lower capacity of proliferation but a more robust antitumor activity compared with the counterpart CD3+CD56- subset, which are seen as precursor cells of CD3+CD56+ cells [33,34]. Consistent with this notion, a significant increase in degranulation was observed in both subpopulations after treatment of 7C6 antibody and CD3+CD56+ cells showed a notably higher degranulation rate than its counterpart. These indicate that engagement of NKG2D with MICA/B is able to trigger and activate both NKT and T cell subpopulations of CIK cells and NKT cells are more sensitive to the NKG2D-MICA/B signaling. However, in other studies [25,35], it was shown that only NK cells rather than CD8 T cells were essential for the therapeutic activity of 7C6 mAb against cancer metastases. Therefore, further in vivo experiments are needed to validate the individual contribution of each CIK subsets in the treatment of 7C6 mAb.

This enhancement of cytolytic activity seems to be more likely due to the increase in surface MICA/B expression, although decrease in shed MICA may also be a relevant contributing factor. Contention remains regarding to the function of soluble NKG2D ligands. Some studies have shown that the existence of soluble MICA/B in the serum could degrade and internalize NKG2D expressed on NK or CD8+ T cells leading to tumor escape from immune response [36]. Others have considered the soluble MICA/B just competitively bound to the NKG2D receptor on these effectors [37]. Opposite to those, soluble Mult1 (a NKG2D ligand exists in mouse) could promote NK cells activation and tumor rejection [38]. One likely explanation is that Mult1 possesses higher affinity than MICA/B to NKG2D receptor. In addition, the downregulation of NKG2D under condition of high level of soluble MICA/B might also be induced by other molecules like transforming growth factor β (TGF-β) [39]. Nevertheless, the downregulation of NKG2D can be reversed by either stimulation with anti-CD3 antibody [36] or cytokines such as IL-2 and IL-15 [40]. Another likelihood is that a single MICA or MICB ligand may not be sufficient to trigger early state of NK or CIK cells as described in an early study [41]. Interestingly, a recent study showed that the α3 domain-specific MICA antibody could completely reverse the soluble MICA-mediated NK cell suppression in a Fc-dependent manner [42]. Regardless, targeting the NKG2D-NKG2D ligand axis has been becoming a promising avenue on the way to fight against malignant diseases.

However, the expression of NKG2D on NK or T cells in some cancer patients was shown to be impaired [36,43] and abnormal NK cell function/activity in patient with different malignancies was also reported [44]. Thus, CIK-based therapy may provide a potent combination partner with MICA/B antibody-mediated immunotherapy.

## 4. Materials and Methods

### 4.1. Cell Lines

MDA-MB-231 breast cancer and Hela cervical cell and K562 leukemia cell were used as targets, cultured in RPMI-1640 (Pan-Biotech, Aidenbach, Bavaria, Germany) medium supplemented with 10% FBS (Sigma-Aldrich Chemie GmbH, Munich, Germany) and 1% penicillin/streptomycin (P/S) (Gibco, Schwerte, Germany) at 37 °C, 5% CO_2_, humidified atmosphere. All the cell lines were purchased from DSMZ (Braunschweig, Germany) and mycoplasma free, as tested by mycoplasma detection kit (Thermo Fisher Scientific, Darmstadt, Germany).

### 4.2. Generation of CIK Cells

CIK cells were generated as previously described [1]. PBMC was isolated from blood of heathy donors (from UKB blood bank) by gradient density centrifugation using Pancoll (Pan-Biotech, Aidenbach, Bavaria, Germany). At the beginning of culture, PBMC was seeded at 3 × 10^6^/mL in a 75 cm^2^ flask for 2 h to remove the monocytes. On day 0, 1000 U/mL IFN-γ (ImmunoTools GmbH, Aidenbach, Bavaria, Germany) was added, followed by the addition of 50 ng/mL anti-CD3 (OKT, eBioscience, Thermo Fisher Scientific, Inc. San Diego, CA, U.S.A.), 600 U/mL IL-2 (ImmunoTools GmbH, Aidenbach, Bavaria, Germany), and 100 U/mL IL-β1 (ImmunoTools GmbH, Aidenbach, Bavaria, Germany) on day 1. Cells were incubated at 37 °C, 5% CO2, humidified atmosphere and subcultured every 3 days in fresh medium supplied with 600U IL-2 at 0.5–1 × 10^6^ cells/mL. After two weeks of ex vivo expansion, CIK cells were collected for experiments.

### 4.3. Antibody Reagents

For flow cytometric analysis, the following antibodies were used: FITC conjugated anti-CD3 (clone OKT3), BV421 conjugated anti-CD8 (clone RPA-T8), PE conjugated anti-CD56 (clone 5.1H11), APC conjugated anti-NKG2D (clone 1D11), APC conjugated anti-MICA/B (clone 6D4) and isotype-matched antibodies were purchased from Biolegend (Koblenz, Germany). APC conjugated anti-CD107a (clone H4A3) and its mouse IgG1 isotype antibody were purchased from BD Biosciences (Heidelberg, Germany).

### 4.4. Phenotypic Analysis

At the end of expansion, CIK cells were harvested to determine phenotype and the expression of NKG2D receptor by flow cytometry (FACS Canto II, BD Biosciences, Heidelberg, Germany). Cells were stained with FITC-CD3, PE-CD56, BV421-CD8, APC-NKG2D and corresponding isotype antibodies. 7AAD (Biolegend, Koblenz, Germany) was added before flow cytometry analysis to gate out viable cells. Samples were acquired using FACS Canto II (BD).

### 4.5. Surface Expression of MICA/B on Tumor Cells

1 × 10^5^ tumor cells per well were cultured in 96-well plates (flat bottom plate for adherent cells, round bottom plate for suspension cells) at 37 °C 5% CO_2_. 7C6 (a human IgG1 mAb kindly provided by Dr Kai W. Wucherpfennig) or human IgG1 isotype control (Biolegend, Koblenz, Germany) antibody were added at 10 µg/mL. After 24 h of culture, MICA and MICB on cell surface were detected following staining with APC conjugated anti-MICA/B antibody or IgG2a isotype control. For detaching adherent cells without disturbing the integrity of surface molecule, Accutase (Biolegend, Koblenz, Germany) was used. Prior to the staining process, Fc receptors were blocked with Human TrueStain FcX™ (Biolegend, Koblenz, Germany) at a final dilution of 1:100. Hoechst 33258 (Cayman Chemical, Hamburg, Germany) was added before flow cytometry analysis for viable cells gating. Samples were acquired using FACS Canto II (BD).

### 4.6. FACS Cytotoxicity Assay

For in vitro cytotoxicity assessment, flow cytometry-based assay was performed as previously described [45] with minor modification. Briefly, 3 × 10^6^ tumor cells were labelled with 0.25µM CFSE (Thermo Fisher Scientific, Eugene, USA) in 1 mL PBS for 20 min at 37 °C in the dark, followed by three times of washing with 5 mL culture medium (containing 10% FBS, to quench the excess CFSE dye). Subsequently, a constant number of cells (5 × 10^4^/well) was co-cultured with CIK cells at different E: T ratios in 96-well plates (flat bottom plate for adherent cells, round bottom plate for suspension cells) at 37 °C, 5% CO_2_. 7C6 or isotype control IgG1 antibody was added at 10 µg/mL at the time of killing assay. For NKG2D blocking experiments, CIK cells were incubated with NKG2D blocking antibody (clone 1D11, Biolegend, Koblenz, Germany) or IgG1 control antibody at 10 µg/mL 1 h before coculture with tumor cells. After 20 h of co-incubation, cells were stained with 7AAD or Hoechst 33258 and measured by FACS Canto II (BD). Accutase was used for the detachment of adherent cells at the end of coculture. In some experiments (K562 and Hela), cytotoxicity was calculated based on the decrease in absolute number of CFSE+ 7AAD− or CFSE+ Hoechst 33258− cells by addition of 5 µL of precision count beads™ (Biolegend, Koblenz, Germany) before FACS analysis. At least 1000 beads were collected in each sample. The following formula was employed for cytotoxicity calculation:

Specific lysis (%) = ((CT − TE)/CT) × 100

CT: absolute number of live CFSE+ tumor cells in control tubes (tumor cells alone); TE: absolute number of live CFSE+ tumor cells in test tubes (tumor + effector).

We then found that there was no difference in specific lysis between bead-based calculation and cell count-based calculation if samples were recorded in a fixed time. Therefore, this method was applied when MDA-MB-231 cells were used as targets. Each sample was collected for 30 s. The lysis formula is modified as below:

Specific lysis (%) = ((CT − TE)/CT) × 100

CT: cell count of live CFSE+ tumor cells in control tubes (tumor cells alone); TE: cell count of live CFSE+ tumor cells in test tubes (tumor + effector).

### 4.7. Degranulation Assay

Degranulation assay was performed using a lysosomal marker CD107a as previously described [46]. Briefly, CIK cells and tumor cells were plated at a 5:1 E/T ratio in the presence of 7C6 or isotype IgG1 antibody at 10 µg/mL in 96 well flat bottom plates. The final volume in each well was 200 µL. Right afterward, 2 µL of APC-conjugated anti-human CD107a antibody was added in each well and incubated for 4 h at 37 °C 5% CO_2_. For NKG2D blocking experiments, CIK cells were incubated with 1D11 antibody or IgG1 control antibody at 10 µg/mL 1 h prior to mixing with tumor cells. After 1 h of E/T coculture, GolgiStop (BD Biosciences, Heidelberg, Germany) was added to each well at a final dilution of 1:1500. At the end of coincubation, cells were washed twice with PBS and stained with FITC-CD3, PE-CD56 and BV421-CD8. Percent CD107a positive cells within the whole population, CD3+CD56− or CD3+CD56+ subpopulation was evaluated using a FACS Canto II (BD).

### 4.8. ELISA Assay

#### 4.8.1. IFN-γ Production by CIK Cells

CIK cells were co-cultured with Hela cells or MDA-MB-231 cells (5 × 10^4^ per well) at a ratio of 20:1 for 24 h in 96-well flat bottom plates without treatment or in the presence of 7C6 or IgG1 isotype antibody at 10 µg/mL. At the end of culture, cell free supernatant was collected for Elisa assay (IFN gamma kit, Invitrogen, Camarillo, CA, USA) following the manufacturer’s instruction.

#### 4.8.2. MICA Shedding by Tumor Cells

3 × 10^4^ of Hela cells and 4 × 10^4^ of MDA-MB-231 cells per well were cultured in 48-well plates with 200 µL complete medium without treatment or in the presence of 7C6 or IgG1 isotype antibody at 10 µg/mL. After 48 h of culture, cell-free supernatant was harvested and the level of soluble MICA was determined using MICA Elisa kit (Duoset DY1300 and DY008, R&D Systems, Inc. Minneapolis, MN, USA) following the manufacturer’s instruction.

### 4.9. Statistical Analysis

FACS data were analyzed using FlowJo V10.6 software (LLC, Ashland, Oregon, USA). Statistical analysis was performed using GraphPad Prism (version 8.0). Experimental data are presented as means ± SD. One-way or two-way analysis of variance (ANOVA) with Bonferroni’s post-hoc test was performed to analyze statistical significance. *p* < 0.05 was considered statistically significant.

## 5. Conclusions

Immunotherapy is showing impressive success in cancer treatment. However, cancer development and progression are complicated processes and regulated by many complex factors. Here we show that MICA-antibody is a specific agent, capable of stabilizing the MICA/B expression on tumor cells and promoting the antitumor activity of CIK cells. The combinatorial therapy of CIK cells with MICA-antibody may provide more benefit for cancer patients and deserves to be further investigated.

## Supplementary Materials

The following are available online at https://www.mdpi.com/2072-6694/12/7/1818/s1, Figure S1: No MICA shed from K562 cells, Figure S2: 7C6 mAb increases the degranulation of CD3+CD56+ NKT cells and CD3+CD56− T cells against K562 cells.
