# Supplementary material for: Increase of Antitumoral Effects of Cytokine-Induced Killer Cells by Antibody-Mediated Inhibition of MICA Shedding
Source: Cancers (Basel). 2020 Jul 7;12(7):1818. doi: 10.3390/cancers12071818 (PMC7408690; doi:10.3390/cancers12071818)
Supplement: Supplementary file 1 [file cancers-12-01818-s001.pdf]

Supplementary Materials

Increase of Antitumoral Effects of Cytokine-Induced Killer Cells by Antibody-Mediated Inhibition of MICA Shedding

Xiaolong Wu, Ying Zhang, Yutao Li and Ingo G.H. Schmidt-Wolf

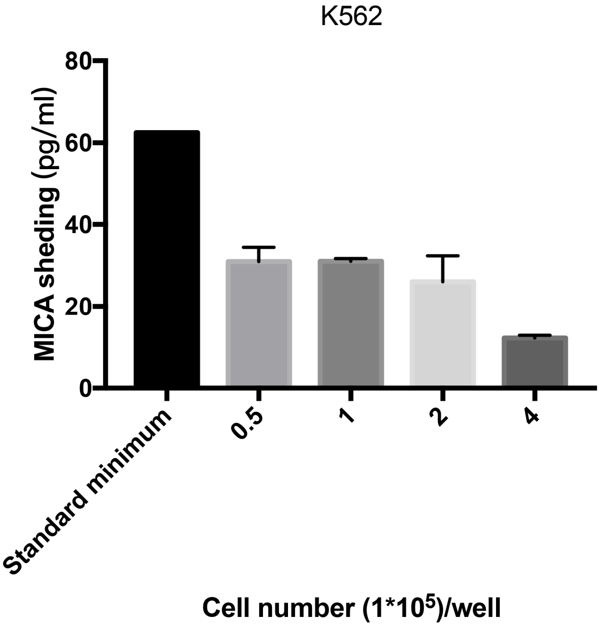

Figure S1. No MICA shed from K562 cells. Indicated number of K562 cells were incubated with 7C6 mAb or IgG1 control antibody at 10 µg/mL for 48 h. Shed MICA was quantified in the supernatant by sandwich ELISA. Data are mean ± SD of duplicate measurements and one representative of three independent experiments. Black bar indicates the sensitivity of this ELISA kit with a minimum detectable concentration of 62.5 pg/mL.

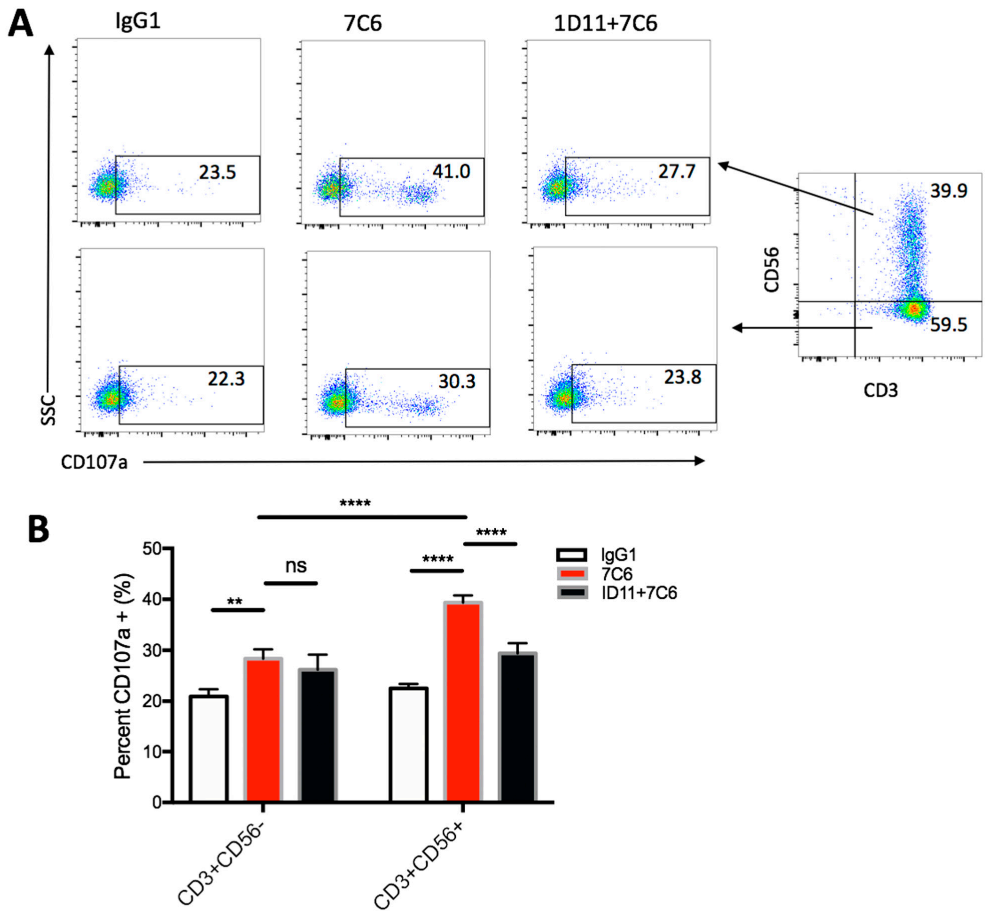

Figure S2. 7C6 mAb increases the degranulation of CD3+CD56+ NKT cells and CD3+CD56- T cells against K562 cells. CIK cells were pretreated with 1D11 or IgG1 antibody at 10 µg/mL 1 h prior to coculture with tumor cells. Afterwards, pretreated CIK cells were coincubated with indicated tumor targets at 5:1 E/T ratio in the presence of 7C6 mAb or IgG1 antibody at 10 µg/mL. APC-CD107a was added at the start of coculture. At the end of 4 h coincubation, degranulation of CIK cells was determined using flow cytometry by staining cells with FITC-CD3 and PE-CD56 antibodies. (**A**) The degranulation of CD3+CD56+ (upper panel) and CD3+CD56- (lower panel) subset cells were analysed. Numbers represent the percentage (%) of gated population. (**B**) Data are mean ± SD of triplicates from 'A', representative of three independent experiments. ns means $p > 0.05$, ** $p < 0.01$, **** $p < 0.0001$ calculated by two-way ANOVA, Bonferroni's post-hoc test.

© 2020 by the authors. Licensee MDPI, Basel, Switzerland. This article is an open access article distributed under the terms and conditions of the Creative Commons Attribution (CC BY) license (http://creativecommons.org/licenses/by/4.0/).
